# Associations of circulation levels of cytokines with birthweight, preterm birth, spontaneous miscarriages, and stillbirth: A Mendelian randomization analysis

**DOI:** 10.3389/fgene.2023.1113804

**Published:** 2023-02-20

**Authors:** Honghong Wang, Jinghang Jiang, Tingwei Jin, Yifu Wang, Mingli Li, Shengzhu Huang, Juanjuan Xie, Zhongyuan Chen, Yi Guo, Jie Zheng, Yonghua Jiang, Zengnan Mo

**Affiliations:** ^1^ Center for Genomic and Personalized Medicine, Guangxi Collaborative Innovation Center for Genomic and Personalized Medicine, Guangxi Key Laboratory for Genomic and Personalized Medicine, Guangxi Medical University, Nanning, Guangxi, China; ^2^ School of Public Health, Guangxi Medical University, Nanning, Guangxi, China; ^3^ Department of Pharmacy, Liuzhou Maternity and Child Healthcare Hospital, Affiliated Maternity Hospital and Affiliated Children’s Hospital of Guangxi University of Science and Technology, Liuzhou, Guangxi, China; ^4^ Department of Pharmacy, Liuzhou Hospital of Guangzhou Women and Children’s Medical Center, Liuzhou, Guangxi, China; ^5^ Institute of Urology and Nephrology, First Affiliated Hospital of Guangxi Medical University, Nanning, Guangxi, China; ^6^ The Reproductive Medicine Center, Jingmen No. 2 People’s Hospital, JingChu University of Technology Affiliated Central Hospital, Jingmen, Hubei, China; ^7^ Guangxi Collaborative Innovation Center for Biomedicine (Guangxi-ASEAN Collaborative Innovation Center for Major Disease Prevention and Treatment), Guangxi Medical University, Nanning, Guangxi, China; ^8^ Department of Gynecology, The Second Affiliated Hospital of Guangxi Medical University, Guangxi Medical University, Nanning, Guangxi, China; ^9^ Life Sciences Institute, Guangxi Medical University, Nanning, Guangxi, China

**Keywords:** Mendelian randomization, cytokines, immune system, birthweight, spontaneous miscarriages, stillbirth, preterm birth

## Abstract

**Background:** The association between immune imbalances and adverse pregnancy outcomes has been extensive investigated by observational studies, but remain unclear. Thus, this study aimed to establish the causality of the circulation levels of cytokines on adverse pregnancy outcomes, such as offspring’s birthweight (BW), preterm birth (PTB), spontaneous miscarriage (SM), and stillbirth (SB).

**Methods:** Two-sample Mendelian randomization (MR) analysis was employed to investigate potential causal relations between 41 cytokines and pregnancy outcomes on the basis of previously published GWAS datasets. Multivariable MR (MVMR) analysis was implemented to investigate the effect of the composition of cytokine networks on the pregnancy outcomes. Potential risk factors were further estimated to explore the potential mediators.

**Results:** Genetic correlation analysis based on large GWAS data sources revealed that genetically predicted MIP1b (*β* = −0.027, S.E. = 0.010, *p* = 0.009) and MCSF (*β* = −0.024, S.E. = 0.011, *p* = 0.029) were associated with reduced offspring’s BW, MCP1 (OR: 0.90, 95% CI: 0.83–0.97, *p* = 0.007) was associated with reduced SM risk, SCF (*β* = −0.014, S.E. = 0.005, *p* = 0.012) associated with decreased number of SB in MVMR. The univariable MR showed that GROa (OR: 0.92, 95% CI: 0.87–0.97, *p* = 0.004) was associated with decreased PTB risk. Except for the MCSF-BW association, all above associations surpassed the Bonferroni corrected threshold. The MVMR results revealed that MIF, SDF1a, MIP1b, MCSF and IP10 composed cytokine networks, associated with offspring’s BW. Risk factors analysis indicated that the above causal associations might be mediated by smoking behaviors.

**Conclusion:** These findings suggest the causal associations of several cytokines with adverse pregnancy outcomes, which were potentially mediated by smoking and obesity. Some of the results did not been corrected through multiple tests and larger samples verification is required in further studies.

## 1 Introduction

Although the world population has increased to over eight billion, reproduction continues to be a relatively low-efficiency and low-flexibility process. The high incidence of implantation failure, spontaneous miscarriage (SM), and stillbirth (SB) shows that adverse pregnancy outcomes are prevalent, resulting in 30%–60% failure in achieving the survival of a clinical progeny (Quenby et al., 2021; Whittaker et al., 1983; Wilcox et al., 1988). Approximately 10.6% of pregnant women in their late pregnancy has preterm birth (PTB) (Chawanpaiboon et al., 2019). Many studies have indicated that children born prematurely had a great risk of neurodevelopmental disorders, chronic lung disease, and behavior problems, which pose a high risk of chronic disease in adulthood (Chawanpaiboon et al., 2019). PTB is a major cause of disease worldwide and challenges health systems and economies (Chawanpaiboon et al., 2019).

The pathogenesis of adverse pregnancy outcomes remains unclear, and effective therapeutic strategy for the prevention of these outcomes remained elusive. To better predict and improve pregnancy outcomes, identifying new sensitive, reliable biomarkers and developing new targeted treatments are crucial. Physiological processes during pregnancy and childbirth are tightly and special. In particular, uterine cavity creates a local environment to protect the embryo from rejection and regulate fetal growth and development. Birth labor is a unique physiological process, in which the terine immune environment shifts immune responses away from immune tolerance state to activation states, leading to myometrial activation (Green and Arck, 2020; Menon, 2022; Meuleman et al., 2015). Thus, the fine balance between fetal and maternal immunological tolerance plays an important role in maintaining normal pregnancy. In recent observational studies, adverse pregnancy outcomes may have been caused by an imbalance of maternal immune tolerance or by direct stimulation by premature or excessive inflammation (Green and Arck, 2020). Aberrant cytokines have been linked to risks of adverse perinatal outcomes, such as SM, PTB, and low birthweight (BW) (Bodnar et al., 2020; Brou et al., 2012; Carp, 2004; Makhseed et al., 2000; Polettini et al., 2017; Sharabi-Nov et al., 2020), however, few Mendelian randomization (MR) analysis have conducted on above causality so far. The results could have been affected by confounding factors, such as smoking, alcohol consumption, underlying disease, and complications, and a correlation was found between findings and many cytokines (Bhat et al., 2013; Brou et al., 2012; Polettini et al., 2017). Nevertheless, population-based studies did not provide robust evidence about the association between cytokines and adverse pregnancy outcomes or how cytokines may impact them.

Two-sample MR analysis is the best source of evidence because of the non-feasibility of randomized controlled trials. MR analysis can benefit from the genetic variants used as instrumental variables of modifiable exposures, eliminating the potential confounds of conventional observational studies. Therefore, we used publicly available large-scale GWAS data. We conducted an MR analysis to investigate 41 kinds of cytokines on adverse pregnancy outcomes (i.e., BW, PTB, SM, and SB). We focused on elucidating the potential causes and effects of adverse pregnancy outcomes to help improve perinatal management, enhance population quality, and provide a new reference for the formulation of maternal and infant health prevention strategies.

## 2 Methods and materials

### 2.1 Data sources

**FIGURE 1:**
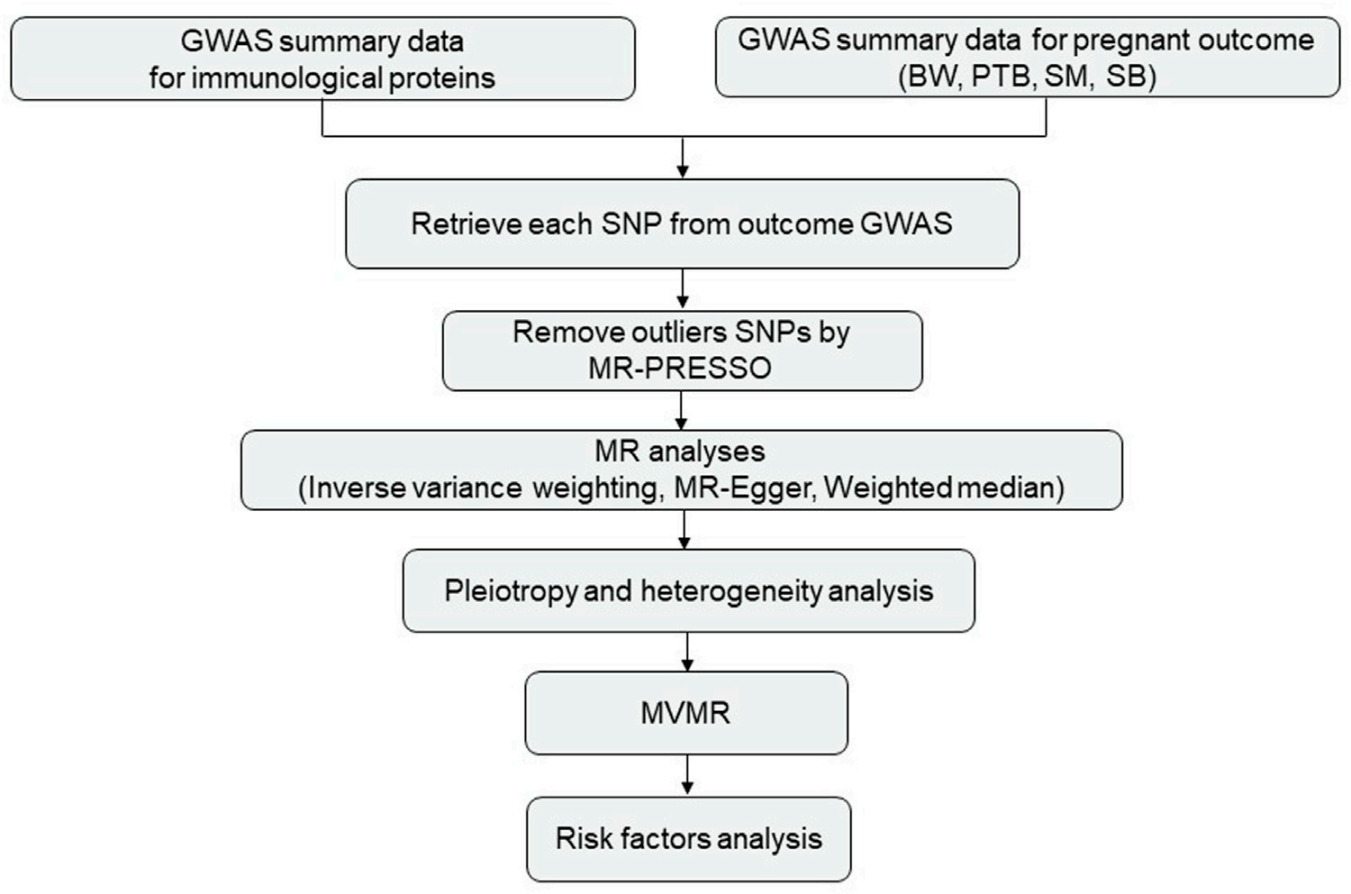
Study design. Abbreviations: BW, birthweight; PTB, preterm birth; SM, spontaneous miscarriage; SB, stillbirth.

The genetic instruments of 41 plasma/serum cytokines were obtained from the IEU GWAS database (https://gwas.mrcieu.ac.uk/) (Ben et al., 2020), which was the most comprehensive and largest meta-analysis consisting of 8,293 individuals from two studies in Finland, the Cardiovascular Risk in Young Finns Study, and FINRISK survey (Ahola-Olli et al., 2017). For the pregnancy outcomes, we obtained summary statistics based on participants of European ancestry by using the offspring’s BW (*n* = 68,258) (Beaumont et al., 2018), the gestational age and PTB (*n* = 1,04,106), SM (*n* = 98,453) and SB (*n* = 78,879) from the IEU GWAS database (https://gwas.mrcieu.ac.uk/) (Ben et al., 2020). PTB and SM from the FinnGen study (https://www.finngen.fi/en). GWAS summary datasets for the number of SBs was obtained from the UK Biobank (http://www.nealelab.is/uk-biobank). Ethical review and informed consent were obtained from the original GWAS. The study design is shown in Figure 1.

### 2.2 Selection of genetic instruments

SNPs associated with the circulation levels of cytokines in the genome-wide significance threshold (*p* < 10^−5^) and independent (10,000 kb pairs apart, r^2^ < 0.001) were selected as instrumental variables (IVs). Approximated F-statistics (beta^2^/se^2^) were applied to evaluate the strength of each genetic instrument (Pierce et al., 2011) and remove the SNP with F-statistics <10 (Supplementary Tables S1, S2). Instruments were then clumped for linkage disequilibrium to help ensure that the SNPs were independent by selecting the SNP with the lowest *p*-value.

### 2.3 MR analyses

Inverse variance weighted (IVW) regression was used as the primary method to obtain the MR effect estimates. We also performed weighted median and MR-Egger regression and compared the results. Cytokines showing evidence derived from univariable MR analysis were considered for multivariable MR (MVMR) analysis, and then other potential cytokines were included for MVMR. Bonferroni correction of the evidential threshold (*p* < 0.05) based on the number of exposures in each analysis. To assess the robustness of the results, we conducted several sensitivity analyses. Cochran’s Q test was used to evaluate heterogeneity among SNPs. MR Egger regression intercept was applied to evaluate the horizontal pleiotropy. MR pleiotropy residual sum and outlier (MR-PRESSO) global test were also performed to assess the presence of pleiotropy and to identify and remove outliers.

### 2.4 Risk factors analysis

To explore the potential mechanisms of association between cytokines and pregnancy outcomes, we further estimated the potential risk factors, such as body mass index (BMI), height, smoking, systolic blood pressure, and type 2 diabetes. Information from the data sources of each risk factor is summarized in the Supplementary Table S3. Cytokines showing evidence derived from univariable MR and MVMR analysis were taken as exposures, and the above potential confounders and risk factors were included as outcomes in the MR analysis. Inverse variance weighted (IVW) regression was used in the primary MR analysis. Then, cytokines derived from MVMR analysis and risk factors were included as exposures, and pregnancy outcome were included as outcomes for MVMR.

### 2.5 Statistical analysis

All analyses were performed using two-sample MR (Version 0.5.6) and MendelianRandomization (version 0.6.0) in R (version 4.0.5).

## 3 Results

### 3.1 Assessment of the association of cytokines with BW, PTB, SM, and SB

#### 3.1.1 Genetically predicted circulation levels of MIP1b, MCSF, SDF1a, and offspring’s BW

**FIGURE 2:**
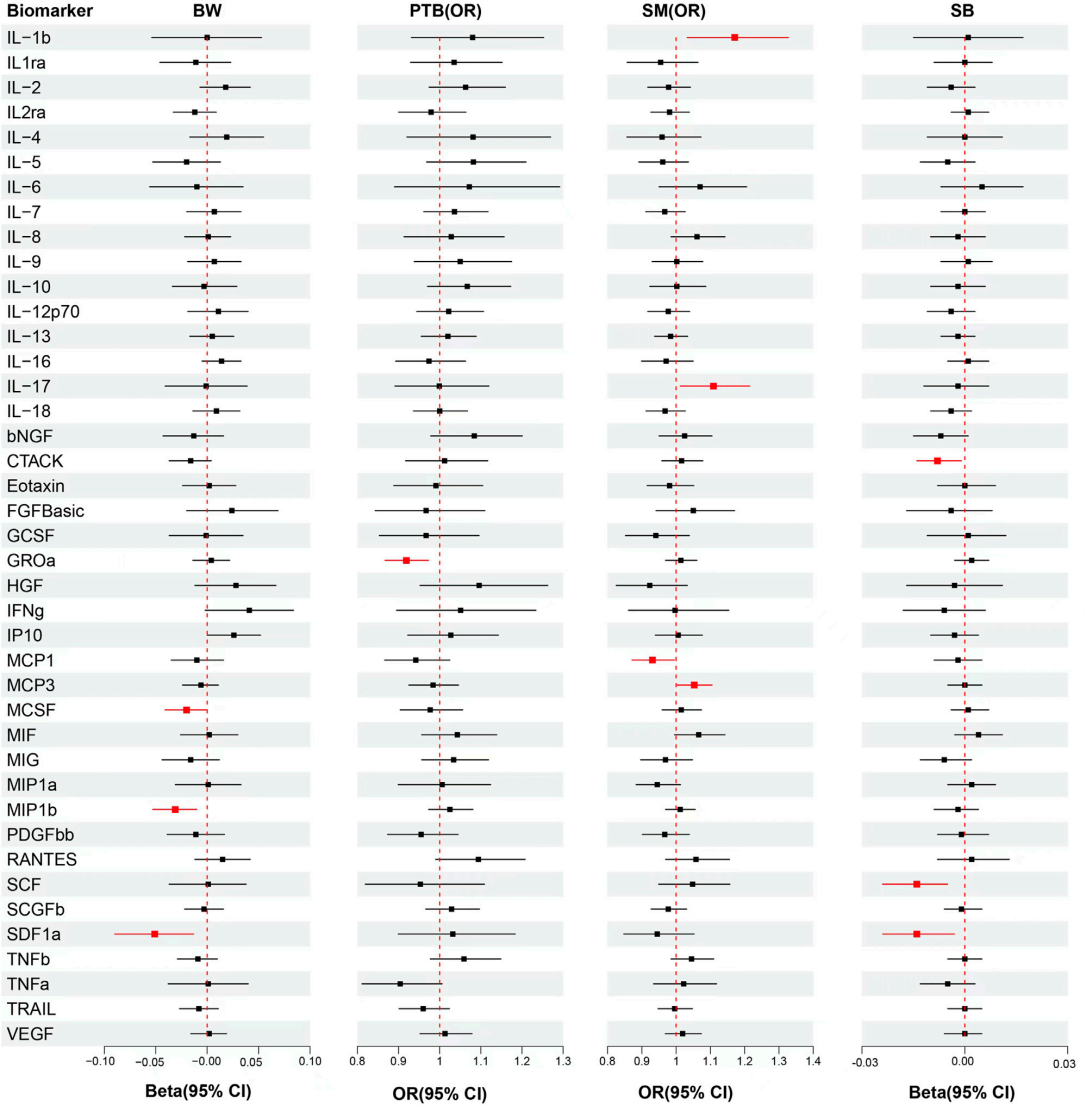
Analytical results from the MR study of the associations between the circulation levels of cytokines and offspring’s BW, PTB, SM, SB. Abbreviations: BW, birthweight; PTB, preterm birth; SM, spontaneous miscarriage; SB, stillbirth; OR, odds ratio; 95% CI: 95% confidence interval. The red lines represented *p* < 0.05.

Univariable MR analysis showed per standard deviation (SD) increased in genetically predicted levels of MIP1b (*β* = −0.031, S.E. = 0.011, *p* = 0.005), SDF1a (*β* = −0.051, S.E. = 0.020, *p* = 0.009) and MCSF(*β* = −0.020, S.E. = 0.010, *p* = 0.046) were associated with reduced offspring’s BW, but each association did not surpass the Bonferroni-corrected *p*-value threshold (Figure 2; Supplementary Table S4).

**FIGURE 3:**
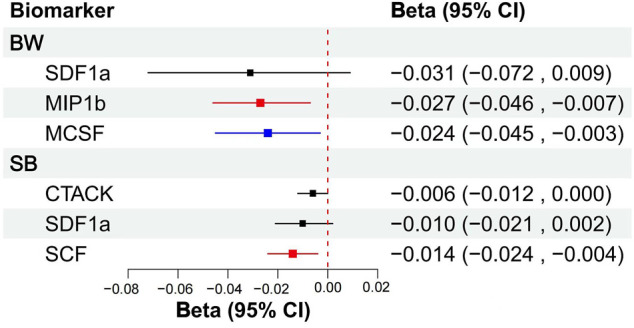
MVMR analytical results of the associations between the circulation levels of cytokines and offspring’s BW, SB. Abbreviations: BW, birthweight; SB, stillbirth; 95% CI: 95% confidence interval. The blue line represented *p* < 0.05, the red lines represented surpassed the Bonferroni-corrected *p*-value threshold.

We included MIP1b, SDF1a, and MCSF in MVMR, the results showed that MIP1b (*β* = −0.027, S.E. = 0.010, *p* = 0.009) and MCSF (*β* = −0.024, S.E. = 0.011, *p* = 0.029) remained associated with offspring’s BW, although only the association of MIP1b surpassed the Bonferroni-corrected *p*-value threshold (*p* < 0.017). After taking into account MIP1b and MCSF, SDF1a (*β* = −0.031, S.E. = 0.021, *p* = 0.136) became non-significant (Figure 3; Supplementary Table S8). Then we try to included other potential cytokines for MVMR, MIF(*β* = −0.030, S.E. = 0.013, *p* = 0.023), SDF1a (*β* = −0.042, S.E. = 0.020, *p* = 0.039), MIP1b (*β* = −0.026, S.E. = 0.009, *p* = 0.007), MCSF(*β* = −0.024, S.E. = 0.011, *p* = 0.030) and IP10 (*β* = 0.047, S.E. = 0.012, *p* = 0.000) composed cytokine networks, which significantly associated with offspring’s BW, but only MIP1b and IP10 surpassed the Bonferroni-corrected *p*-value threshold (*p* < 0.008) (Supplementary Table S10).

#### 3.1.2 Genetically predicted circulation levels of GROa and PTB risk

The results from univariable MR showed that each SD raised in genetically predicted levels of GROa (OR: 0.92, 95% CI: 0.87–0.97, *p* = 0.004) decreased PTB risk, but the association did not surpass the Bonferroni-corrected *p*-value threshold (*p* < 0.003) (Figure 2; Supplementary Table S5).

Then, we try to included other potential cytokines for MVMR. After taking into account GROa, increased Eotaxin (OR: 0.92, 95% CI: 0.86–0.99, *p* = 0.035) levels was associated with attenuated risk for PTB, but GROa (OR: 1.02, 95% CI: 0.93–1.12, *p* = 0.634) did not remain significant associated with PTB risk (Supplementary Table S11). The association of Eotaxin did not surpass the Bonferroni-corrected *p*-value threshold (*p* < 0.025).

#### 3.1.3 Genetically predicted circulation levels of MCP1, MCP3, IL-17, IL-1b, and SM risk

In univariable MR analysis, each SD increased in genetically predicted levels of MCP1 (OR: 0.93, 95% CI: 0.87–1.00, *p* = 0.037) was associated with decressed SM risk. However, raised MCP3 (OR: 1.05, 95% CI: 1.00–1.11, *p* = 0.038), IL-17 (OR: 1.11, 95% CI: 1.01–1.21, *p* = 0.026) and IL-1b (OR: 1.17, 95% CI: 1.03–1.33, *p* = 0.014) were associated with increased SM risk, but each association did not surpass the Bonferroni-corrected *p*-value threshold (Figure 2; Supplementary Table S6).

**FIGURE 4:**
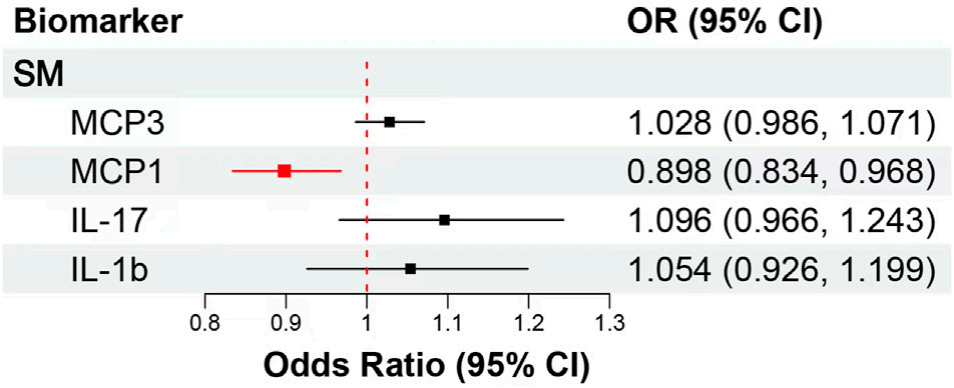
MVMR analytical results of the associations between the circulation levels of cytokines and SM. Abbreviations: SM, spontaneous miscarriage; 95% CI: 95% confidence interval. The red lines represented surpassed the Bonferroni-corrected *p*-value threshold.

We included MCP1, MCP3, IL-17, and IL-1b in MVMR. After taking into account MCP3, IL-17 and IL-1b, raised MCP1 (OR: 0.90, 95% CI: 0.83–0.97, *p* = 0.007) remained associated with decreased risk of SM, and the association surpassed the Bonferroni-corrected *p*-value threshold (*p* < 0.013), but MCP3 (OR: 1.03, 95% CI: 0.99–1.07, *p* = 0.197), IL-17 (OR: 1.10, 95% CI: 0.97–1.24, *p* = 0.161) and IL-1b (OR: 1.05, 95% CI: 0.93–1.20, *p* = 0.433) did not remain significant associated with SM risk (Figure 4; Supplementary Table S9). Then, we try to included other potential cytokines for MVMR, after taking into account MCP1, MCP3, IL-17, and IL-1b, raised IL-8 (OR: 1.18, 95% CI: 1.07–1.31, *p* = 0.002) and RANTES (OR: 1.10, 95% CI: 1.02–1.18, *p* = 0.017) were significantly associated with increased SM risk, but only IL-8 surpassed the Bonferroni-corrected *p*-value threshold (*p* < 0.008) (Supplementary Table S11).

#### 3.1.4 Genetically predicted circulation levels of SCF, CTACK, SDF1a, and SB

Univariable MR **s**howed that each SD increase in genetically predicted levels of SCF (*β* = −0.014, S.E. = 0.005, *p* = 0.004), CTACK (*β* = −0.008, S.E. = 0.003, *p* = 0.025) and SDF1 (*β* = −0.014, S.E. = 0.005, *p* = 0.012) were associated with decreased number of SB, but each association did not surpass the Bonferroni-corrected *p*-value threshold (Figure 2; Supplementary Table S7).

Then, MVMR was performed to analyze whether CTACK, SCF, and SDF1a were associated with the risk of SB. After taking into account CTACK and SDF1a, raised SCF (*β* = −0.014, S.E. = 0.005, *p* = 0.012) remained associated with decreased number of SB, and the association surpassed the Bonferroni-corrected *p*-value threshold (*p* < 0.017), but CTACK (*β* = −0.006, S.E. = 0.003, *p* = 0.062) and SDF1a (*β* = −0.010, S.E. = 0.006, *p* = 0.104) did not remain significant associated with number of SB (Figure 3; Supplementary Table S8). Then, we try to included other potential cytokines for MVMR, no significant association was observed for other cytokines.

### 3.2 Sensitivity analyses

#### 3.2.1 Analysis of horizontal pleiotropy

The MR-Egger regression intercept test showed that no evidence of horizontal pleiotropy (all *p >* 0.05) (Supplementary Table S12). MR-PRESSO indicated that horizontal pleiotropy may affected analyses of Eotaxin, MIF with BW; bNGF with PTB; and GCSF and MIG with SB (all *p* ≤ 0.05). Following outlier correction, there was no evidence for the association above (all *p* > 0.05) (Supplementary Table S13).

#### 3.2.2 Analysis of heterogeneity

In addition, in-depth analyses showed a significant disparity in the heterogeneity between cytokines and the following pregnancy outcomes: PTB and CTACK, Eotaxin; SM and IL1ra; SB and MIG, MIP1b, RANTES. None of the other cytokines showed significant heterogeneity with adverse pregnancy outcomes (all *p* > 0.05) (Supplementary Table S12).

### 3.3 Analysis of risk factors

The potential mediators between cytokines and adverse pregnancy outcomes were further explored. Univariable MR **s**howed that each SD increase in genetically predicted levels of SDF1a (*β* = 0.006, S.E. = 0.003, *p* = 0.025), MCP3 (*β* = 0.003, S.E. = 0.001, *p* = 0.039) associated with increased smoking; raised GROa (*β* = −0.006, S.E. = 0.003, *p* = 0.047), IL-17 (*β* = −0.013, S.E. = 0.006, *p* = 0.044) associated with decreased BMI; IL-1b (*β* = 0.016, S.E. = 0.005, *p* = 0.003), RANTES (*β* = −0.007, S.E. = 0.003, *p* = 0.029) associated with height, but only IL-1b associated with height surpassed the Bonferroni-corrected *p*-value threshold (*p* < 0.007) (Supplementary Table S14).

The MVMR results suggested that smoking behaviors might be responsible for MIF (*β* = −0.029, S.E. = 0.013, *p* = 0.035), MIP1b (*β* = −0.023, S.E. = 0.010, *p* = 0.028), MCSF(*β* = −0.031, S.E. = 0.011, *p* = 0.007), IP10 (*β* = 0.044, S.E. = 0.013, *p* = 0.001) linked offspring’s BW, but only MCSF and IP10 association surpassed the Bonferroni-corrected *p*-value threshold (*p* < 0.008). Smoking behaviors also might be potential mediators linking for GROa (OR: 0.91, 95% CI: 0.85–0.98, *p* = 0.010) linked risk of PTB, MCP1 (OR: 0.89, 95% CI: 0.82–0.96, *p* = 0.003) and IL-8 (OR: 1.19, 95% CI: 1.07–1.33, *p* = 0.003) linked SM risk, and SCF (*β* = −0.012, S.E. = 0.005, *p* = 0.031) linked number of SB, all above associations surpassed the Bonferroni corrected threshold. The SDF1a-BW (*β* = 0.053, S.E. = 0.016, *p* = 0.001) association enhanced after taking into account body mass index in MVMR, and surpassed the Bonferroni corrected threshold (Supplementary Table S15).

## 4 Discussion

MR analysis was conducted to investigate whether cytokines were causally associated with BW, PTB, SM, and SB and to identify causative relations between cytokine and pregnancy outcomes. The circulation levels of MIP1b, MCSF, GROa, MCP1, SCF and involved complex cytokines network were associated with the risk of adverse pregnancy outcomes, which might be potentially mediated by smoking behaviors and BMI.

During pregnancy, the maternal immune system is reprogrammed to tolerate allogeneic (paternal) fetal antigens, in which a stepwise progression of tolerance is clearly observed. As a result of creating immune imbalances, pregnancy outcomes are jeopardized (Green and Arck, 2020; Menon, 2022). Given that cytokines influence the pregnancy outcomes, this result could have significant clinical implications (Dai et al., 2022). First, cytokine is not only useful as a tool for identifying a high-risk pregnancy but also for stratifying risk groups. Second, immunotherapy may provide for adverse pregnancy outcomes with a novel treatment option.

Numerous observational cohort studies had linked the levels of the biomarkers MIP1b, MCSF, GROa, Eotaxin, MCP3, and SCF in the regulation of embryo implantation (Gnainsky et al., 2010), improve endometrial receptivity (Gnainsky et al., 2015), premature rupture of membranes (Raba et al., 2021), recurrent miscarriage (Wu et al., 2021), reduced developmental potential of an oocyte (Tan et al., 2017), and gestational hypertension (Lan et al., 2022). IP10 has been found to promote the differentiation and migration of T cells toward Th1 and Th17 cells. This phenomenon contributes to the construction of the local proinflammatory environment of endometrial tissue and facilitates tissue remodeling and angiogenesis. Such processes can enhance the implantation of embryos based on the association with the endometrial microenvironment during the first trimester (Gervasi et al., 2012; Jia and Li, 2020; Jiang et al., 2021). MCP-1, RANTES might be predictive factors for diabetic pregnancy in early pregnancy development, which levels associated with perinatal outcome (Wender-Ozegowska et al., 2008). These findings were strongly supported by our results.

Maternal immune imbalances might be associated with a range of negative experiences, such as infection, smoking (Morales-Prieto et al., 2022; Prins et al., 2012), exposure to air pollutants (Buxton et al., 2019), symptoms of anxiety, depression, and other mood disorders (Camacho-Arroyo et al., 2021), and promotion of inflammation directly or indirectly by the reproductive tract (Kumar et al., 2021) or gut microbiota dysbiosis (Chen et al., 2019), that contribute to pregnancy outcomes. During pregnancy, changes to a woman’s physiologic state of lungs are reasons for the mother’s increased susceptibility to infectious agents. A rapid cellular and humoral immune response is evoked during infection, increasing the possibility of suffering from adverse pregnancy outcomes. Patients infected with influenza (Uchide et al., 2012), COVID-19 (Rosen et al., 2022), Zika virus (Rabelo et al., 2020), dengue virus (Nunes et al., 2019), *Chlamydia trachomatis* of the reproductive tract (Faris et al., 2019), HIV (Akoto et al., 2021; Morrison et al., 2018), *Plasmodium falciparum* (Chêne et al., 2014; Dobaño et al., 2018), and latent tuberculosis (Indrati et al., 2022) had elevated levels of MIP1b, GROa, IL-8, RANTES, and IP10, but reduced levels of Eotaxin, MCP-1 (Requena et al., 2015), associated with the inflammation in the placenta. The serum cytokine expression levels, which were associated with the type of infection, could provide predictive information about pathogen infection, mother-to-child-vertical transmission (Kumar et al., 2012; Schnittman et al., 2021), immunity protection of host (Amaral et al., 2020), and the risk of adverse pregnancy outcomes.

Maternal smoking affects functional development of placenta, and constrain the delivery of nutrients and oxygen. However, tobacco induced maternal-fetal immunoregulatory changes during pregnancy have not been studied deeply (Morales-Prieto et al., 2022). The current findings may suggest new insights into the relationship between smoking behaviors, cytokines network, and adverse pregnancy outcomes.

In addition, given that adverse pregnancy outcomes had multiple causes, secreted cytokines apparently induce a more complex network rather than act independently with maternal response. Therefore, the complex network played a role in influencing the fetal development and function. Multiple biomarkers might have a better prognostic/predictive relevance. Bodnar et al. (2020) described a unique neurodevelopmental delay atlas, suggesting that cytokine imbalance may have their origin in the neurodevelopmental disorders of a child. The effectiveness of a comprehensive risk biomarker for predicting preterm deliveries could be enhanced than a single risk factor. In addition, an apparent discrepancy among different races had been proposed (Bhat et al., 2013; Brou et al., 2012). We estimated the effects of the cytokine network on pregnancy outcomes using multivariable MR, which have not been studied deeply, and further evidence is still needed to confirm it.

Although robust study protocols and strict implementation had been performed, this study still presents some limitations. First, larger sample sizes are required for GWAS discovery to generalize conclusions. Second, genetic variants that represented a cumulative lifetime exposure were incorporated into the MR, and a larger cohort study is necessary to confirm our findings. Finally, the dissimilar findings in MR may have been caused by separate regions and differences in genetics and environment among the diverse ethnic groups.

In conclusion, disturbances in cytokine homeostasis, immune imbalances, and adverse pregnancy outcomes correlations of causality could be estimated from exposure and outcome data with the aid of more comprehensive GWAS datasets and two-sample MR study. An evaluation of the effects of cytokine networks on pregnancy outcomes was initiated in this study by using MVMR. Inflammation cells were activated abnormally and collaborated subtly, resulting in adverse perinatal outcomes. The significant clinical implications for studying the molecular mechanisms of immune imbalance-mediated adverse pregnancy outcomes and identifying high-risk maternal individuals and immunotherapy were discussed. The results could present various implications for policies and programs at the prepotency level. Independent validation studies and prospective cohort studies will be necessary to verify the conclusion.

## Data Availability

The original contributions presented in the study are included in the article/Supplementary Material, further inquiries can be directed to the corresponding author.
